# Prenatal Sonographic and Molecular Genetic Diagnosis of Popliteal Pterygium Syndrome

**DOI:** 10.3390/diagnostics11101819

**Published:** 2021-10-01

**Authors:** Kuntharee Traisrisilp, Suchaya Luewan, Sirinart Sirilert, Phudit Jatavan, Theera Tongsong

**Affiliations:** Department of Obstetrics and Gynecology, Faculty of Medicine, Chiang Mai University, Chiang Mai 50200, Thailand; kuntharee.t@cmu.ac.th (K.T.); suchaya.l@cmu.ac.th (S.L.); sirinart.s@cmu.ac.th (S.S.); kod.thanata@gmail.com (P.J.)

**Keywords:** popliteal pterygium syndrome, popliteal webbing, prenatal diagnosis, ultrasound

## Abstract

Popliteal pterygium syndrome (PPS) is an extremely rare autosomal dominant disorder, characterized by the cleft palate with or without cleft lip, limbs abnormalities with highly characteristic features of popliteal webbing, syndactyly, and genital abnormalities and nail anomalies. Prenatal diagnosis of PPS has been extremely rare. We describe a unique case of fetal PPS at 20 weeks of gestation. The diagnosis of PPS was based on the ultrasound findings of bilateral popliteal webbings, extending from posterior aspects of the upper thighs through the lower legs, resulting in restriction in knee extension, bilateral equinovarus feet with syndactyly, ambiguous genitalia and the grooved lip. Anatomical structures were otherwise normal. Trio whole-exome sequencing revealed a de novo heterozygous *IRF6* gene mutation in the fetus, confirming the diagnosis with PPS. In conclusion, popliteal webbing or combination of facial cleft or cleft variants and bilateral abnormal postures of the lower limbs is suggestive of PPS and genetic diagnosis should be warranted.

## 1. Introduction

Popliteal pterygium syndrome (PPS) is an autosomal dominant disease with highly variable expressivity and incomplete penetrance. PPS is associated with a mutation of the *IRF6* gene, localized to chromosome 1q32.2 [1,2]. Its occurrence is extremely rare. The true prevalence is not known but is estimated to be approximately 1 in 300,000 live births [3,4], with more than 200 cases being reported worldwide. PPS is characterized by a spectrum of anomalies involving orofacial, cutaneous, musculoskeletal, and genital anomalies. The minimal diagnostic criteria for PPS are any three of the following cleft lip/palate, popliteal pterygium, paramedian lower lip sinuses, genital anomalies and toenail abnormalities, especially syndactyly [5]. Popliteal pterygium involves webbing of the skin extending from the ischial tuberosities to the heels, resulting in severe malposition of the lower limbs. Abnormal genitalia are very common, particularly bifid scrotum and cryptorchidism in males and hypoplasia of the labia majora and uterus in females. Since the disease can be associated with severe morbidity, though most have good prognosis, early prenatal diagnosis is essential for counseling and guiding management. Prenatal diagnosis is possible by means of genetic sequencing of the *IRF6* gene in DNA extracted from the fetal samples obtained by chorionic villi sampling or amniocentesis, or provisional diagnosis by fetal ultrasound. The prognosis is generally good, with normal mental development and the possibility to correct most of the alterations through targeted surgery. Nearly all of the cases in previous reports are postnatally diagnosed. To the best of our knowledge, only few cases were prenatally detected [6,7]. The objective of this study is to describe prenatal ultrasound features of popliteal pterygium syndrome, in de novo cases of the fetuses, which was confirmed the diagnosis by trio whole-exome sequencing.

## 2. Case Presentation

This case study was ethically approved by the Institutional Review Boards (Faculty of Medicine, Chiang Mai University, Thailand; Ethic Research ID: 8272/Study Code: OBG-2564-08272).

**Figure 1:**
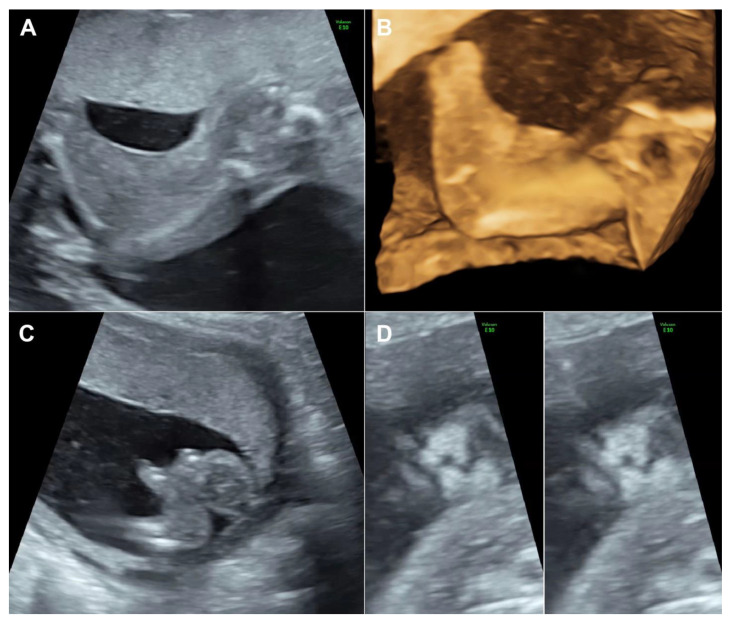
(**A**) Webbing from the posterior aspect of the thigh extending to the heels; (**B**) 3D-US reconstruction of the lower limb shows popliteal webbing; (**C**) Club foot shows ectrodactyly; (**D**) Coronal view of the fetal face shows bilateral grooves of the upper lip (left: superficial plane; right: deeper plane).

**Figure 2:**
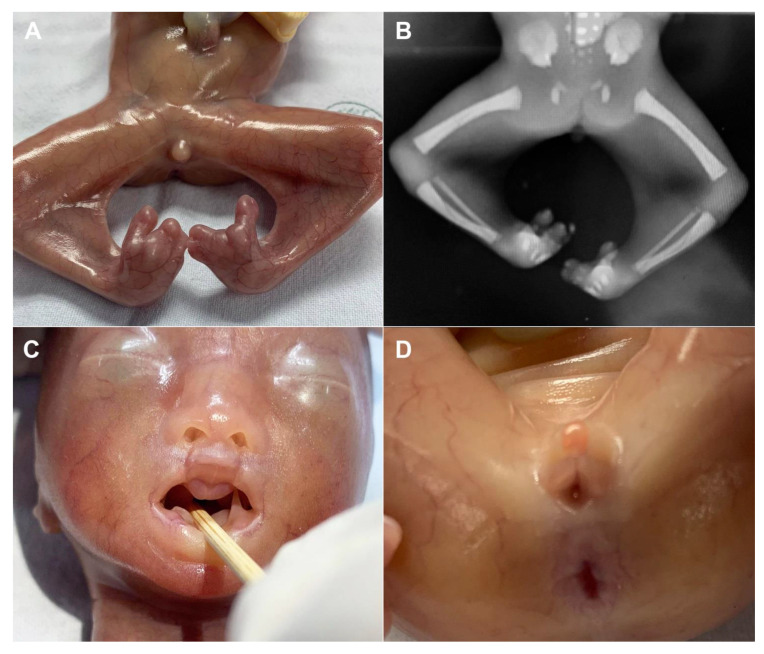
(**A**) Postnatal findings of the popliteal webbings and syndactyly/ectrodactyly; (**B**) X-ray of the lower limbs (abnormal postures); (**C**) Postnatal findings of the grooves in the upper lip and intraoral soft tissue adhesion; (**C**) Ambiguous genitalia (poorly developed scrotum with hypospadias).

A 30-year-old pregnant woman, G3 P1011, presented for antenatal care and fetal anomaly screening at 20 weeks of gestation. She had no serious underlying medical disease, except left traumatic optic neuropathy. Also, her familial history was unremarkable. The first pregnancy ended up with miscarriage at 8 weeks of gestation. The second pregnancy gave birth to a healthy term female newborn, weighing 3550 g. The current pregnancy course was uneventful. Ultrasound examination for fetal anomaly screening at 20 week of gestation showed a single viable fetus with abnormalities as follows (Figure 1): bilateral popliteal pterygia extending from posterior aspect of the thighs to the heels with restriction in extension of both knees (no more than 90°), bilateral talipes equinovarus, syndactyly and ectrodactyly of the toes, ambiguous genitalia (penis-like phallus with poorly developed scrotum and suspected of hypospadias) and suspicious of cleft lips (variant). Biometry was consistent with gestational age (using Voluson E10 machine equipped with transabdominal 2- to 4-MHz curvilinear transducers; GE Healthcare Ultrasound, Milwaukee, WI, USA). The placenta was normal and amniotic fluid volume was normal. Both upper limbs were structurally normal and had normal movement. No other structural anomaly was identified. Fetal cord blood which was obtained by cordocentesis and parental blood samples were sent for trio whole-exome sequencing. Because of the severe abnormalities of both lower extremities and genital structures, the couple requested to have the pregnancy terminated. Therapeutic abortion was performed using transvaginal misoprostol. A male fetus with ambiguous genitalia was aborted at the 20 weeks of gestation, weighing 420 g. Postnatal findings confirmed the prenatal sonographic features (bilateral popliteal webs extending from posterior aspect of the upper thighs to the heels with bilateral talipes equinovarus, syndactyly and ectrodactyly of the toes, and ambiguous genitalia), as presented in Figure 2. The placenta was grossly normal. Fetal karyotype was 46, XY. The molecular genetic analysis by whole-exome sequencing was subsequently performed. All exon regions of all human genes were captured by xGen Exome Research Panel v2 (Integrated DNA Technologies, Coralville, IA, USA). The captured regions of the genome were sequenced with Novaseq 6000 (Illumina, San Diego, CA, USA). The raw genome sequencing data analysis, including alignment to the GRCh37/hg19 human reference genome, variant calling and annotation, was conducted with open-source bioinformatics tools and in-house software (3 billion Inc. Seoul, Seoul, Korea). The result revealed a heterozygous pathogenic variant in *IRF6* (NM_0061474; c.250C > T; p.Arg84Cys) in the fetus, consistent with molecular diagnosis of Popliteal Pterygium Syndrome type 1 (MIM #119500). This condition is inherited by autosomal dominant manner. However, trio analysis in this family proves that the pathogenic variant in the affected case occurred de novo. Therefore, the recurrent risk in the next pregnancy is less than 1%. This information was provided to the couples.

## 3. Discussion

To the best of our knowledge, prenatal diagnosis of PPS is very rarely reported. We have searched for prenatal diagnosis of PPS on PubMed, EMBASE, and CINAHL. Only two case reports were found. The first case with prenatal diagnosis was reported by Perrotin et al. in 2000 [7], on the basis of the main sonographic findings of severe amyotrophy with fixed knees, equinovarus feet and cleft lip/palate as well as postnatal confirmation of bilateral popliteal pterygia. However, no molecular genetic diagnosis was performed. In 2014, Posey et al. [6] reported a prenatal diagnosis of PPS with ultrasound (cleft) and MRI (popliteal web) at 24 weeks of gestation and postnatal confirmation with *IRF6* mutation analysis. The summary of prenatal cases is presented in Table 1

Postnatally, abnormalities commonly seen in PPS are as follows [8]: facial clefts (91–97%), lower lip pits or sinuses (45%), popliteal pterygium (webbing) with some degree of contractures or restriction of mobility (58%), syndactyly (30%), genitourinary anomalies (37%), and skin abnormalities around the nails (33%). Other occasional features include syngnathia, missing teeth, spina bifida occulta, and retrognathia. Nevertheless, the prevalence of those findings may be different in prenatal life. The cleft lip is readily identified with prenatal ultrasound but cleft palate without cleft lip or other subtle anomalies mentioned above can simply be missed in routine anomaly screening. However, based on our cases and previous case reports [6,7], cleft lips, though just shallow grooves of the lip seen in our case, seem to be the first clue for further searching, leading to pattern recognition of the syndrome and molecular genetic testing. In cases of high risk on the basis of familial history (case reported by Perrotin et al.), only one abnormality should warrant genetic confirmation, while, in the cases of de novo like our case, pattern relatively specific for PPS may be needed for molecular genetic testing. Based on the case presented here and previous case reports, pattern recognition for prenatal diagnosis includes (1) facial clefts, (2) bilateral popliteal webbing with impaired mobility together with abnormal postures of lower limbs, especially equinovarus and syndactyly, and (3) ambiguous genitalia. Such a sonographic pattern should warrant genetic diagnosis of PPS, even in cases of no familial history. Molecular genetic testing may be accomplished by chorionic villous sampling, especially in cases of known mutation in the parents, amniocentesis, or umbilical cord blood sampling. PPS is strongly associated with missense mutations in the DNA binding domain of *IRF6* (encoded in exons 3 and 4) that alter residues that are expected to interact directly with DNA.

The case presented here emphasizes on careful delineation of anatomical survey. Abnormal posture of the lower limbs including clubfeet (equinovarus), restriction of lower limb movement was the first clue, warranting detailed ultrasound. Actually, the well-defined popliteal pterygia can be simply missed, if not exactly midsagittal scans of the lower limbs. It is noteworthy that cross-sectional scans, oblique scans or coronal scans along the long axis view of the lower limbs cannot clearly demonstrate the popliteal webbing. The examiner must carefully identify the true midsagittal scans to clearly visualize the popliteal webbing as presented in Figure 1 and Video 1. In cases of high suspicion, 3D-ultrasound can be helpful in reconstruction to demonstrate the webbing (Figure 2). On literature review, popliteal webbing is very rarely demonstrated clearly with prenatal ultrasound. Nevertheless, since the sonographic images of extremities can be visualized in early gestation, it is, theoretically, possible that the popliteal webbing can be detected earlier in late first trimester or early second trimester. However, the diagnosis in our case, unfortunately, was detected at 20 weeks of gestation, relatively late, since we routinely perform anomaly screening at mid-pregnancy, while first trimester screening is only optional, not routine.

The main differential diagnoses include Van der Woude syndrome (VWS), a disorder caused by deletions and mutations in the same gene (*IRF6*) and is the most common generic form of syndromic orofacial cleft [9]. In fact, affected individuals in the same family, having the same mutation in *IRF6*, have been diagnosed with PPS and with VWS. The cause of this variable expressivity is not known. The underlying genetic mechanism that results in a different effect of IRF6 function is hypothesized (haploinsufficiency for VWS, missense mutation for PPS). Other genetic modifiers and environmental factors are purposed [1]. Bartsocas-Papas syndrome (recessive form of PPS), CHAND syndrome and multiple pterygium syndrome should also be considered.

On genetic counseling, the recurrence risk of PPS as an autosomal dominant disorder, in the subsequent pregnancy is approximately 50%. Careful physical examination of family members is emphasized due to subtle malformation such as lower lip pits, fold of skin overlying the nail may not be discovered before, incomplete penetrance as well as variable expressivity including intra-familial variability of this syndrome [10]. If PPS has been identified in the family, prenatal diagnosis should be considered, through mutational analysis of the fetal samples obtained by amniocentesis or chorionic villus sampling. In de novo mutations, as reported in this case, the recurrent rate should be extremely low. The prognosis is generally good. Growth and mental development are expected to be normal. However, the prognosis for physical disabilities depends on the severity of the pterygium, genital abnormalities and orofacial defect. Many cases need multiple corrective surgeries that might burden patients and family’s quality of life. Genital anomalies may cause infertility. Accordingly, the options of pregnancy termination in cases of early detection may be offered.

## 4. Conclusions

Since PPS can be associated with severe morbidity, prenatal diagnosis is essential. We describe the prenatal diagnosis of PPS, with first prenatal document of de novo *IRF6* mutations. The prenatal pattern recognition of PPS includes facial clefts, popliteal webbing with abnormal postures of lower limbs and ambiguous genitalia. Facial clefts and abnormal lower limbs seem to be sensitive markers, whereas popliteal webbing is a specific marker. With 2D-US, popliteal webbing is best demonstrated in midsagittal scans of the lower limbs and 3D-US can be supportive. Based on this case report and previous studies, molecular genetic diagnosis of PPS may be considered in cases of familial history (disease in one of the parents), particularly in the presence of any abnormality in the sonographic pattern of PPS, or in cases of no familial history but meeting the following ultrasound features: (1) a facial cleft together with bilateral abnormal posture of the lower limbs, or (2) popliteal webbing.

## Figures and Tables

**Table 1:** Summary of prenatal diagnosis of popliteal pterygium syndrome (PPS) with postnatal/abortal associations.

Report	GA at Prenatal Diagnosis	Main Ultrasound/MRI Findings	Familial History	Outcomes and Postnatal/Abortal Findings	Molecular Genetic Diagnosis
Perrotin et al. [7] 2000	18 weeks	Bilateral cleft lips, equinovarus, leg amyotrophy, foot deformity, fixed knee, ambiguous genitalia	Positive (Clinical diagnosis in the mother)	TOP at 21 weeks, additional finding: intraoral syngnathia, popliteal pterygia	Not done
Posey et al. [6] 2014	24 weeks	IUGR, syngnathia, cleft lip, bilateral popliteal pterygia, equinovarus, syndactyly, small scrotum, duplicating renal collecting system (MRI)	No	CS, 35 weeks, weight 1.87 Kg, additional finding: ankyloblepharon, genital anomaly, micro/retrognathia, syngnathia	*IRF6* mutation in newborn (parents not done)
This study	19 weeks	Grooved lips, bilateral popliteal pterygia, ambiguous genitalia, syndactyly/ectrodactyly	No	TOP at 20 weeks additional finding: intraoral web, cleft soft palate	*IRF6* de novo mutation(Trio WES)

## Data Availability

The data of this report are available from the corresponding authors upon request.
